# Hollow-Architected Heteroatom-Doped Carbon-Supported Nanoscale Cu/Co as an Enhanced Magnetic Activator for Oxone to Degrade Toxicants in Water

**DOI:** 10.3390/nano13182565

**Published:** 2023-09-15

**Authors:** Tran Doan Trang, Jia-Yin Lin, Hou-Chien Chang, Nguyen Nhat Huy, Suresh Ghotekar, Kun-Yi Andrew Lin, Venkata Subbaiah Munagapati, Yeoh Fei Yee, Yi-Feng Lin

**Affiliations:** 1 Department of Environmental Engineering & Innovation, Development Center of Sustainable Agriculture, National Chung Hsing University, Taichung 402, Taiwan; 2 Semiconductor and Green Technology Program, National Chung Hsing University, 250 Kuo-Kuang Road, Taichung 402, Taiwan; 3 Department of Chemical Engineering, National Chung Hsing University, Taichung 402, Taiwan; 4 Faculty of Environment and Natural Resources, Ho Chi Minh City University of Technology (HCMUT), Ho Chi Minh City 700000, Vietnam; nnhuy@hcmut.edu.vn; 5 Vietnam National University Ho Chi Minh City, Ho Chi Minh City 700000, Vietnam; 6 Centre for Herbal Pharmacology and Environmental Sustainability, Chettinad Hospital and Research Institute, Chettinad Academy of Research and Education, Kelambakkam 603103, Tamil Nadu, India; ghotekarsuresh7@gmail.com; 7 Institute of Analytical and Environmental Sciences, National Tsing Hua University, Hsinchu 300, Taiwan; 8 Research Centre for Soil & Water Resources and Natural Disaster Prevention (SWAN), National Yunlin University of Science and Technology, Douliou 64002, Taiwan; 9 School of Materials and Mineral Resources Engineering, Engineering Campus, Universiti Sains Malaysia, Nibong Tebal 14300, Penang, Malaysia; 10 Department of Chemical Engineering and R&D Center for Membrane Technology, Chung Yuan Christian University, 200 Chung Pei Rd., Chungli, Taoyuan 320, Taiwan

**Keywords:** peroxymonosulfate, sulfate radical, AOPs, MOFs, alloy

## Abstract

Even though transition metals can activate Oxone to degrade toxic contaminants, bimetallic materials possess higher catalytic activities because of synergistic effects, making them more attractive for Oxone activation. Herein, nanoscale CuCo-bearing N-doped carbon (CuCoNC) can be designed to afford a hollow structure as well as CuCo species by adopting cobaltic metal organic frameworks as a template. In contrast to Co-bearing N-doped carbon (CoNC), which lacks the Cu dopant, CuCo alloy nanoparticles (NPs) are contained by the Cu dopant within the carbonaceous matrix, giving CuCoNC more prominent electrochemical properties and larger porous structures and highly nitrogen moieties. CuCoNC, as a result, has a significantly higher capability compared to CoNC and Co_3_O_4_ NPs, for Oxone activation to degrade a toxic contaminant, Rhodamine B (RDMB). Furthermore, CuCoNC+Oxone has a smaller activation energy for RDMB elimination and maintains its superior effectiveness for removing RDMB in various water conditions. The computational chemistry insights have revealed the RDMB degradation mechanism. This study reveals that CuCoNC is a useful activator for Oxone to eliminate RDMB.

## 1. Introduction

Wet oxidation technologies (WOTs) would be practical for eliminating organic pollutants, particularly contaminants from textile industries. Conventionally, WOTs are associated with production of radicals that exhibit strong redox powers to decompose contaminants. As the Fenton’s reaction is commonly employed for ^•^OH to degrade pollutants [1], SO_4_^•−^-related WOTs would receive increasing attention since SO_4_^•−^ exhibits a notably higher potential [2,3,4], making SO_4_^•−^-related WOTs appealing to eliminate organic pollutants.

To produce SO_4_^•−^, a few reagents would be accessible, such as Oxone^®^, which, however, requires activation for quickly releasing SO_4_^•−^ from Oxone. Amid several activating strategies [5,6,7,8], heterogeneous catalysis is considered to be a practical approach [3,4]. For instance, Hu et al. studied Oxone activation using CoMg/SBA-15 for eliminating Rhodamine B (RDMB) [9]. Co_x_Fe_3−x_O_4_ nanocatalysts were also reported to activate Oxone for the removal of RDMB [10]. Likewise, several metallic materials had been also reported for activating Oxone to degrade RDMB [11,12].

Even though Co has been validated as the most efficient metal and a few cobaltic materials (e.g., Co_3_O_4_) have been fabricated for activating Oxone [13,14], recent studies reported that dual-metal substances, comprising Co with other metals, would possess additional benefits that could enhance capabilities [15,16]. Particularly, dual-metal Co-bearing alloys would exhibit enhanced performances because of more active sites [17], and the combination of Cu and Co species seem to be useful dual-metal catalysts for activating Oxone [17,18,19] as Cu is abundant and possesses superior redox properties.

However, the employment of CuCo alloy nanoparticles (NPs) might encounter challenges, including the aggregation of NPs, inconvenient recovery of NPs, and vulnerable instability [20,21]. Thus, immobilizing CuCo NPs into supports is a useful technique.

On the other hand, as carbon is the most Earth-abundant, highly accessible, and stable, carbon materials would be favorable media for supporting/embedding CuCo NPs [22,23]. Carbonaceous media are also modified by nitrogen to increase active sites [24,25]. Thus, fabricating a material by immobilizing CuCo on N-doped carbon (NC) media seems promising for activating Oxone.

Even though embedding NPs onto carbon media is feasible by additional fabrication, such an additional fabrication strategy unfortunately faces many issues, including uneven distribution, agglomeration, and insufficient loading of active species [26,27]. Adopting templates that consist of desired materials (e.g., metal and carbon precursors) appears to be a convenient method to obtain such a composite after the carbonization of such templates. Specifically, zeolitic imidazolate frameworks (ZIFs) would be a useful precursor because of cobaltic constituents and nitrogenic ligands [28] that would be transformed to NC. Doping Cu into ZIF could further lead to the formation of CuCo.

While Co-ZIF has a cubic geometry and sub-micron sizes, the derived Co/carbon hybrids are quite challenging to access by Oxone [11,29]. Thus, here, we present a convenient strategy to afford so-called hollow-structured Co-ZIF using an etching technique to remove its interior, forming a hollow configuration. Following doping Cu on such a hollow material and then carbonization, a nanoscale CuCo species-bearing NC (CuCoNC) is then created. This CuCoNC comprising CuCo bimetallic NP embedded in NC appears to be a useful catalyst for activating Oxone to eliminate toxic contaminants. In particular, Rhodamine B (RDMB) is then selected as a model toxic organic pollutant as RDMB is a carcinogenic pollutant [30,31].

To further examine the catalytic activities of CuCoNC, a Co-bearing N-doped carbon (CoNC), which is an analogous to CuCoNC without the Cu dopant, was also prepared.

## 2. Experimental

**Figure 1:**
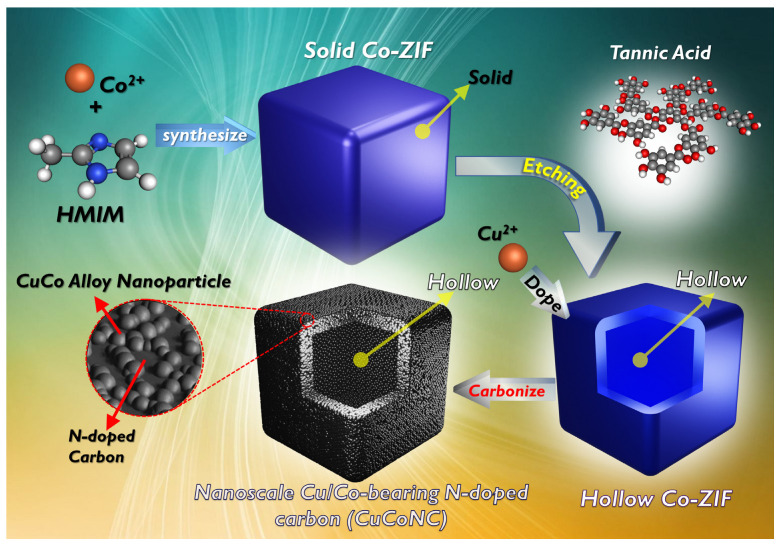
Preparation scheme for CuCoNC.

The fabrication of CuCoNC was implemented as depicted in Figure 1, and the detailed synthesis process, RDMB degradation by Oxone, analytical procedures, and computational chemistry are provided in the supplementary material.

For calculating the elimination rate constant, the following equation was adopted for the estimatiation of *k*:

*Ln*(*C_t_*/*C*_0_) = −*kt* (1)

## 3. Results and Discussion

### 3.1. Properties of CuCoNC

***Morphologies and Compositional analyses***

**Figure 2:**
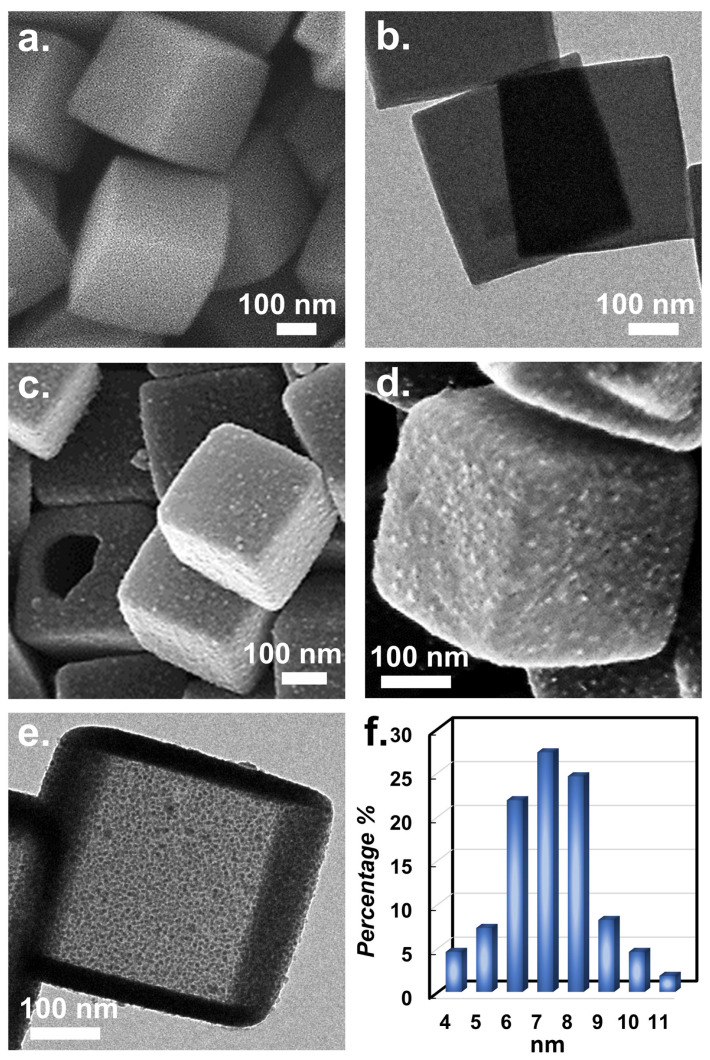
Images of (**a**,**b**) solid Co-ZIF; (**c**–**e**) hollow Co-ZIF; and (**f**) size distribution of NPs in CuCoNC.

**Figure 3:**
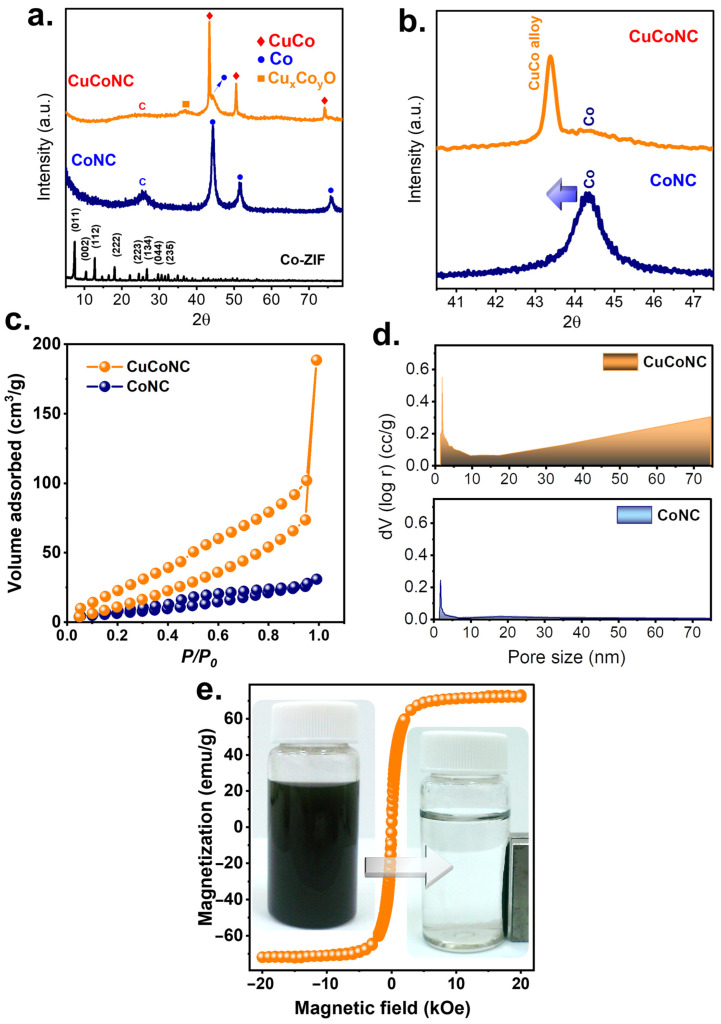
(**a**,**b**). Crystalline structures; (**c**). adsorption, (**d**) pores of CuCoNC and CoNC; (**e**) magnetism of CuCoNC.

Since CuCoNC was derived from Co-ZIF, the appearance of Co-ZIF was firstly characterized as displayed in Figure 2a, revealing the cubic morphology with flat faces and clearly defined edges. The transmission electron microscopic image of Co-ZIF (Figure 2b) demonstrates that the pristine Co-ZIF was solid without any holes/voids. Figure 3a shows that the XRD result of pristine Co-ZIF was consistent with the literature [11,32,33], confirming that Co-ZIF was successfully formed.

Via modification by tannic acid as well as Cu^2+^, and then pyrolysis, the resulting material (Figure 2e) maintained the cubic shape; nevertheless, the planes of the resultant material became noticeably coarse and decorated by many NPs. Moreover, the solid structure changed to a configuration with pores, as observed by the rupture. Its transmission electron microscopic image (Figure 2f) further validated that the solid texture of pristine Co-ZIF disappeared and was substituted by a hollow structure. The formation of a hollow structure could be attributed to the addition of tannic acid which releases free proton (H^+^), permeating into Co-ZIF, eventually affording a hollow structure. Moreover, the transmission electron microscopic image also indicated that the NPs decorated ranged from 4 to 11 nm, demonstrating that these NPs were extremely small and the sizes were relatively similar.

Moreover, Figure 2e shows that the resulting material was comprised of numerous NPs. Therefore, the solid Co-ZIF was successfully changed, exhibiting a different XRD result (Figure 3a).

**Figure 4:**
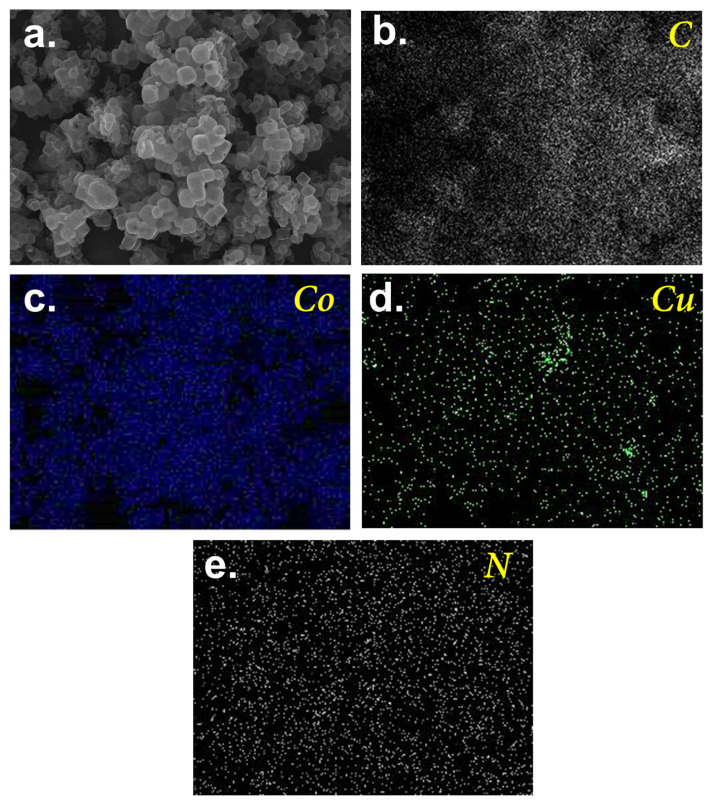
Mapping analysis of CuCoNC: (**a**) scanned region, (**b**) C, (**c**) Co, (**d**) Cu, and (**e**) N.

In particular, a few peaks were observed at 25.6°, 37.0°, 43.4°, 44.3°, 50.5°, and 74.1°. While the broad peak at 25.6° could be attributed to carbon, the noticeable peaks at 43.4, 50.5, and 74.1° would be ascribed to the formation of CuCo [34] as a result of the spinel Co structure being substituted by Cu, leading to the peak shift towards the lower value of 2θ as shown in Figure 3b [34]. Nevertheless, a peak of Co (111) could still be noted at 44.3°, suggesting that a fraction of Co remained in the resulting material. Moreover, the minor existence of Cu/Co oxide was also observed at 37.0°, possibly due to the surficial oxidation of CuCo species upon exposure to air. Figure 4 further displays the elemental analysis of this resulting product, in which notable signals of C, N, Co, and Cu were observed. While most elements can be well-distributed over the carbon matrix, in the case of the distribution of Cu, some localization in the intercubic spaces can also be observed. This was possibly because Cu was doped onto Co-ZIF through mixing Cu and Co-ZIF, and such a phenomenon of localization possibly occurred.

These results suggest that the resultant NPs adorned on the hollow cubic product were comprised of Cu/Co species, formulating the nanoscale Cu/Co-bearing N-doped carbon (CuCoNC).

A similarity to CuCoNC was also fabricated in the absence of Cu^2+^ dopant in Figure S1a,b with only noticeable signals of Co and C in the absence of Cu (Figure S1c). Figure 3a also displays the XRD pattern of this resulting product from Co-ZIF without Cu^2+^, which contained peaks at 25.6°, 44.3°, 51.6°, and 75.9°. While the peak at 25.6° could be ascribed to carbon, the rest of the peaks can be well-indexed to Co^0^. Thus, the ZIF without Cu^2+^ dopant became Co-bearing N-doped carbon (CoNC) after the etching and carbonization treatments.

***Textural properties***

Figure 3c further shows its adsorption result which could be categorized as an IUPAC IV sorption with a notable hysteresis loop, indicating that CuCoNC exhibited mesopores. Simultaneously, Figure 3c also reveals the N_2_ sorption of CoNC, which was actually similar to that of CuCoNC but with a much smaller N_2_ sorption volume. the surface area of CuCoNC was 70 m^2^/g, while CoNC merely showed a surface area of 26 m^2^/g. Figure 3d also reveals the pore size distribution of CuCoNC, in which a large fraction of pores in the mesoscale range could be observed, and the total pore volume was 0.29 cc/g, whereas the total pore volume of CoNC volume was merely 0.045 cc/g. The relatively superior and complicated textural properties in CuCoNC were possibly owing to the fact that the faces of CuCoNC exhibited noticeable small holes as seen in Figure 3d and the extremely small spherical NPs were decorated on the surface, leading to much more roughened surfaces. In contrast, the faces of CoNC were smooth and flattened without noticeable bumps and holes on the surface as seen in Figure S1. Such a difference also suggests that the Cu dopant into the Co-ZIF seemed to alter the texture by introducing hetero-atoms, thereby causing the greater surface area.

It would be interesting to examine the magnetism of CuCoNC. In Figure 3e, CuCoNC exhibited a significantly high magnetism approaching 72 emu/g, which enabled CuCoNC to be easily controllable for uniform dispersion and quick collection as shown in the inset.

### 3.2. Degradation of RDMB by CuCoNC+Oxone

**Figure 5:**
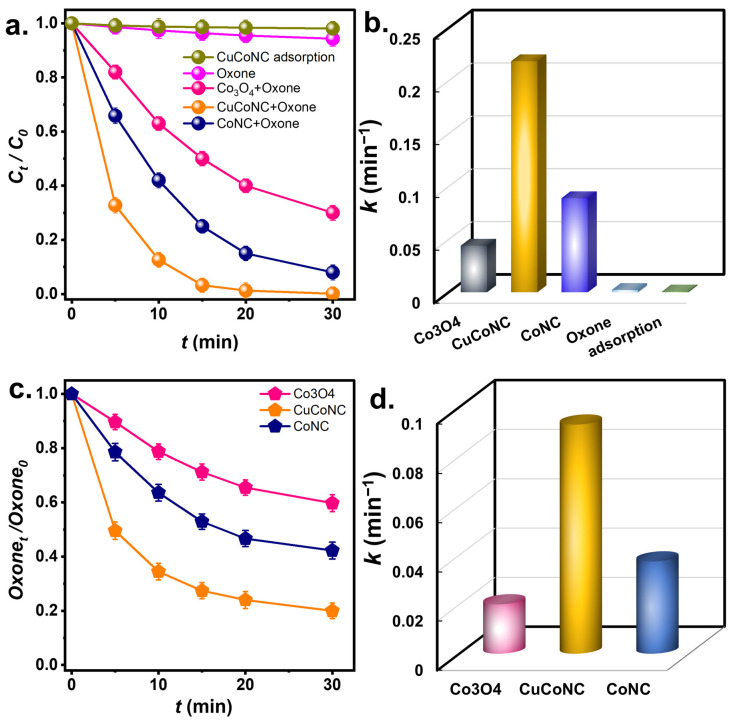
(**a**) RDMB elimination (initial concentration of RDMB = 10 mg/L) and (**b**) kinetics; (**c**) Oxone decomposition and (**d**) kinetics of Oxone consumption (Catalyst = 50 mg/L, Oxone = 100 mg/L, T = 30 °C).

The degradation of RDMB by Oxone using CuCoNC was then measured in Figure 5. Because RDMB might be removed by sorption, the sorption of RDMB on CuCoNC was then studied. However, RDMB barely decreased using CuCoNC, and RDMB was not eliminated by sorption. On the other hand, by using Oxone alone, RDMB was only slightly degraded in 30 min. Nevertheless, when CuCoNC and Oxone were adopted simultaneously, RDMB dropped quickly in 30 min, indicating that CuCoNC activated Oxone to eliminate RDMB. The commercial Co_3_O_4_ (Figure S2) and CoNC were also employed to activate Oxone to eliminate RDMB in Figure 5a. Co_3_O_4_+Oxone enabled *C_t_/C_0_* to reach 0.32 after 30 min, while CoNC+Oxone led to *C_t_/C*_0_ = 0.07.

In addition, the rate constants for RDMB degradation are then summarized in Figure 5b. The rate constant (*k*) for CuCoNC+Oxone would be 0.219 min^−1^, which was greater than the rate constants for Co_3_O_4_ NP+Oxone (i.e., 0.043 min^−1^) and CoNC+Oxone (0.089 min^−1^), indicating that CuCoNC was certainly superior to the commercial Co_3_O_4_ NP and CoNC.

As CuCoNC led to the much faster degradation of RDMB, it was interesting to study Oxone decomposition by CuCoNC. In Figure 5c, the consumption of Oxone during RDMB degradation was analyzed. In 30 min, >80% of Oxone was consumed by CuCoNC, whereas Oxone consumptions by CoNC and Co_3_O_4_ NP were significantly lower (40% and 55%, respectively). K values of Oxone decomposition are also summarized in Figure 5d. The *k* using CuCoNC was 0.092 min^−1^, the *k* by CoNC was 0.037 min^−1^, and that by Co_3_O_4_ NP was 0.020 min^−1^, confirming that the Oxone decomposition by CuCoNC was significantly faster and more efficient than CoNC and Co_3_O_4_ NP, thereby causing faster RDMB degradation.

**Figure 6:**
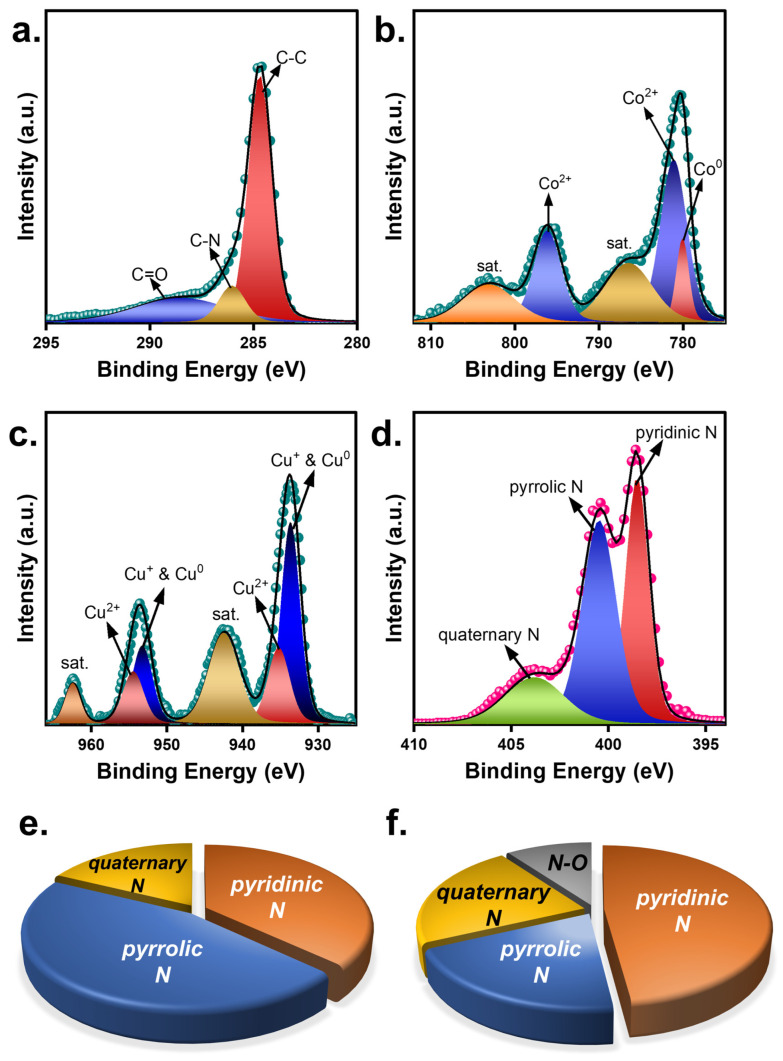
(**a**–**d**) XPS results of CuCoNC; nitrogen groups in (**e**) CuCoNC; (**f**) CoNC.

Oxone activation correlates to surficial chemistry between Oxone and catalysts [35]. Since CuCoNC showed a significantly superior capability for activating Oxone, it was intriguing to investigate these catalysts. In Figure 6, X-ray photoelectron spectroscopy (XPS) results of CuCoNC and CoNC are displayed. In particular, the C1s result of CuCoNC (Figure 6a) is decomposed to several peaks at 283.9, 286.5, and 288.6 eV, corresponding to the C-C, C-N, and C=O groups [36]. In addition, the Co2p result of CuCoNC would be deconvoluted to a series of peaks (Figure 6b). In particular, the peak at 778.7 eV was Co^0^, whereas the peaks at 780.3 and 796.2 eV were Co^2+^ [37]. Moreover, the bands at 933.0 and 953.2 eV correspond to Cu^0^ and Cu^+^ in CuCo alloy [38]; the bands at 935.2 and 954.4 eV can be ascribed to Cu^2+^ species, as the oxidation of CuCo can occur in contact with O_2_ as seen in the XRD pattern. Furthermore, Figure 6d shows many peaks at 398.2, 399.3, and 400.6 eV, attributed to the pyridinic N, pyrrolic N, and quaternary N group [39].

Similarly, the N1s in CoNC (Figure S3) also had pyridinic N, pyrrolic N, quaternary N, and the N-O group, respectively [39]. Despite the fact that both CuCoNC and CoNC consisted of Co species, CuCoNC additionally contained Cu^0^, Cu^+^, and Cu^2+^, that act as additional mediating groups for Oxone activation [40,41].

In addition, CuCoNC also showed a different result for nitrogen components, with pyridinic N, pyrrolic N, and quaternary N of 37.0%, 45.3%, and 17.7%, respectively (Table S1), while CoNC showed pyridinic N, pyrrolic N, quaternary N, and N-O of 48.1%, 19.9%, 22.1%, and 9.9%, respectively. As pyridinic N and pyrrolic N are confirmed as highly effective for Oxone activation [42], the total quantity of these N species in CuCoNC (i.e., 82.3%) was much larger than in CoNC (68.0%), thereby promoting catalytic activities of CuCoNC for activating Oxone in RDMB decomposition.

**Figure 7:**
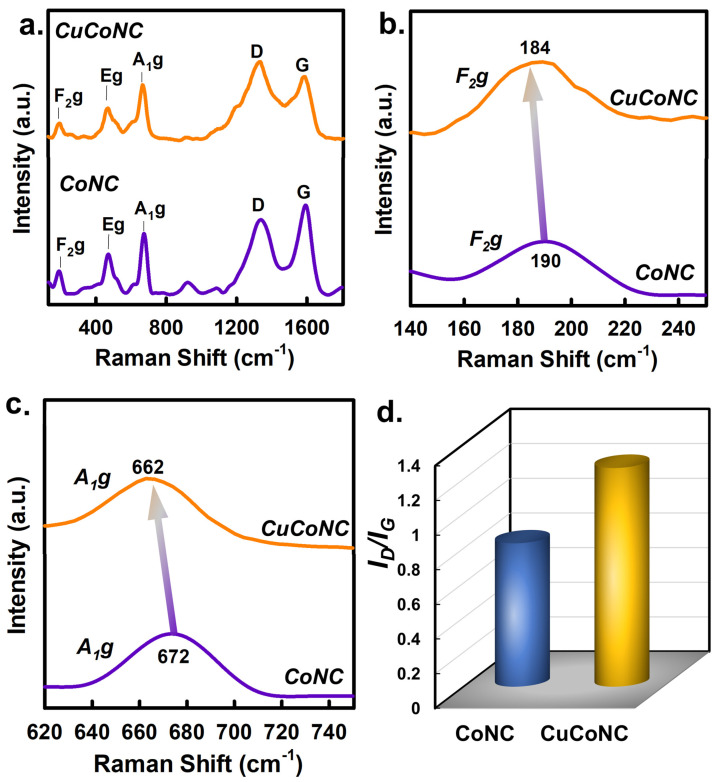
(**a**) Raman analysis of CuCoNC and CoNC; (**b**,**c**) regional result of CuCoNC and CoNC; and (**d**) G and D peaks.

Figure 7a displays the Raman result of CuCoNC and CoNC; both showed similar bands at around 190, 470, and 670 cm^−1^, corresponding to *F*_2_*g*, *E_g_*, and *A*_1_*g* [43]. As CuCoNC and CoNC were composed of similar patterns, the zoomed-in images of these bands unraveled differences. Specifically, the *F*_2_*g* peak of CoNC was located at 190 cm^−1^ (Figure 7b), but the peak of CuCoNC changed to 184 cm^−1^. The peak variance could also be seen in the case of *A*_1_*g* (Figure 7c). The *A*_1_*g* peak of CoNC was located at 672 cm^−1^, and the peak in CuCoNC changed from 672 to 662 cm^−1^. As *A*_1_*g* and *F*_2_*g* are relevant to the coordination of Co species, these changes implied that the CuCoNC seemed to comprise more defects or oxygen vacancies [44]. The higher fraction of D band in CuCoNC (Figure 7d) (Table S2) might also be the reason for CoNC exhibiting higher catalytic activities [45].

**Figure 8:**
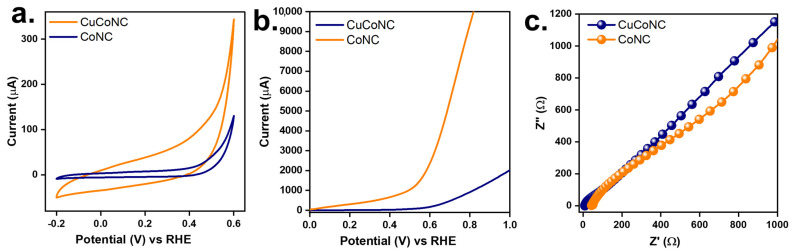
Electrochemical properties of CuCoNC and CoNC: (**a**) CV curves, (**b**) LSV curves, (**c**) EIS Nyquist plots.

These differences might also allow CuCoNC to exhibit more reactive sites [44], thereby enhancing the activation of Oxone. Moreover, Oxone activation would also be associated with redox properties [35]. Figure 8a displays the cyclic voltammetry (CV) curves of CuCoNC and CoNC. CoNC showed a smaller CV loop, while CuCoNC revealed a larger CV loop. CuCoNC possessed 14.0 F/g, which was larger than that of CoNC as 3.3 F/g, demonstrating that CuCoNC showed superior redox properties.

Moreover, the linear sweep voltammogram (LSV) result of CuCoNC is shown in Figure 8b. CuCoNC required an initial potential of 0.50 V but CoNC required a slightly higher initial potential of 0.82 V to reach 0.1 mA. This result demonstrated that CuCoNC was superior to CoNC for electrical transport. Figure 8c further shows that CuCoNC possessed a slightly narrower semi-circle in the high-frequency region than CoNC, revealing that CuCoNC possessed a larger extent of charge transfer [46].

### 3.3. RDMB by CuCoNC+Oxone: Other Effects

**Figure 9:**
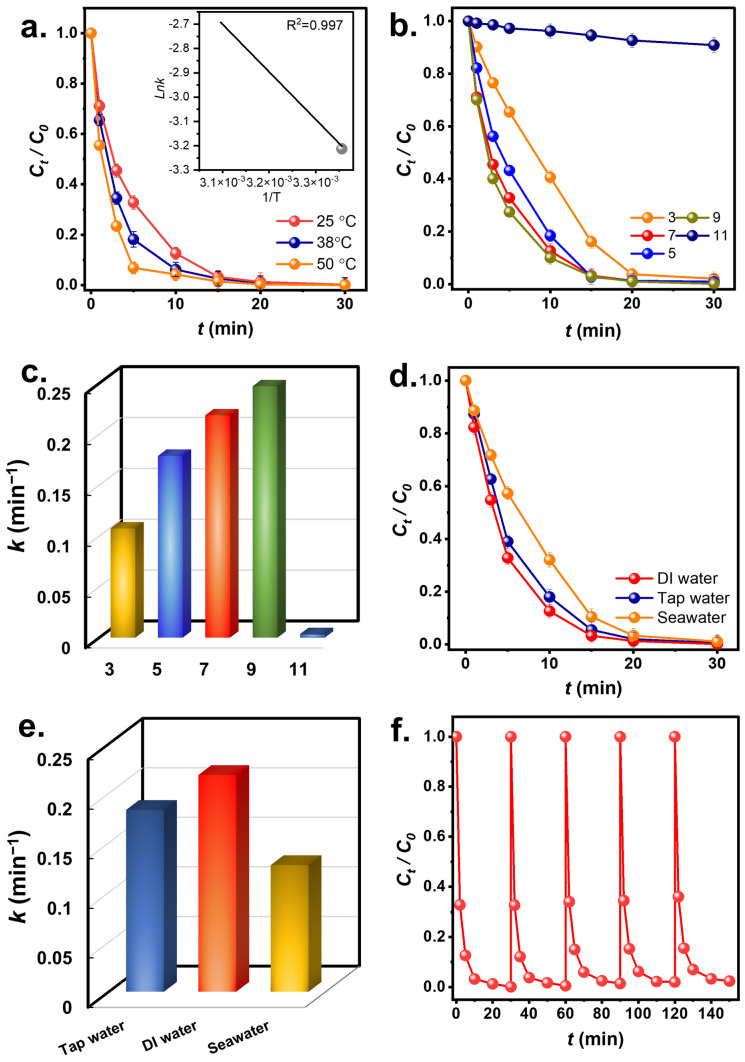
(**a**) Temperature effect, (**b**,**c**) pH effect and corresponding k, (**d**,**e**) scavenger effect; (**f**) reusability.

As CuCoNC+Oxone eliminated RDMB effectively, temperature and pH would also impact RDMB degradation. Figure 9a shows RDMB eradication by CuCoNC+Oxone at 25 to 50 °C. As the temperature rose from 25 to 38 °C, the k value for RDMB degradation rose from 0.219 min^−1^ to 0.329 min^−1^. When it further rose to 50 °C, the rate of RDMB degradation increased to 0.520 min^−1^, indicating the favorable effect of elevated temperatures on RDMB elimination. Furthermore, these rate constants at different temperatures were correlated using the Arrhenius equation [47]:

(2) $$\ln k=\ln A-\frac{E_{a}}{RT}$$

The corresponding *E_a_* was 27.7 kJ/mol, much lower than the reported *E_a_* values of RDMB elimination in the literature (Table S3). Additionally, Table S4 also comprehensively compares CuCoNC with other reported catalysts for RDMB degradation. These comparisons further demonstrate that CuCoNC was a superior activator for Oxone to degrade RDMB.

In Figure 9b, the pH effect was then studied. At pH = 7, RDMB was degraded quickly; nevertheless, at pH = 5, the k dropped to 0.178 min^−1^. At pH = 3, RDMB degradation was even slower with *k* = 0.107 min^−1^. H^+^ under these acidic conditions might react with radicals to “destroy” them as follows: [48]:

H^+^ + SO_4_^•−^ + e^−^ → HSO_4_^−^ (3)

H^+^ + ^•^OH + e^−^ → H_2_O (4)

Furthermore, Oxone tends to become sluggish in acidic environments [49], thus resulting in less effective RDMB degradation. As the solution was alkaline at pH = 9, RDMB elimination seemed to remain the same with a slightly increased rate constant of 0.247 min^−1^. This might be because RDMB is a cationic molecule, the surface charge of CuCoNC became relatively more negative as shown in Figure S4. Therefore, the electrostatic attraction between RDMB and CuCoNC might grow stronger, facilitating RDMB degradation. At pH = 11, the *k* considerably dropped to *k* = 0.004 min^−1^. This result also demonstrates that the strongly alkaline condition would cause unfavorable effects on RDMB degradation [49].

In addition to DI water, tap water and seawater would be also employed as water matrices in Figure 9d. With tap water, the beginning of RDMB elimination with *k* = 0.183 min^−1^ was still very efficient. Tap water may contain impurities but the RDMB elimination was quite effective, as RDMB was still eliminated. In the case of seawater, the lowered *k* value of 0.123 min^−1^ was obtained as seawater contains a large amounts of substances and impurities, such as Cl^-^, which has been validated to interfere with Oxone [50].

### 3.4. Reusability Test

Since CuCoNC can efficiently eliminate RDMB, the reusability of CuCoNC to eliminate RDMB over multiple rounds was examined. Figure 9f shows that CuCoNC could eliminate RDMB over 5 rounds. This result validates that CuCoNC retained its effectiveness for Oxone activation in RDMB elimination. The XRD analysis of spent CuCoNC (Figure S5) shows that it remained almost comparable, demonstrating that CuCoNC would be a durable activator.

### 3.5. Mechanistic Studies by CuCoNC+Oxone

For further unraveling the degradation mechanism, a few probe agents were then employed for elucidating ROS during RDMB degradation. At first, *t*-butyl alcohol (TBA) was used to be a probe agent for ^•^OH, while MeOH would be used to identify presence of both SO_4_^•−^ and ^•^OH. After TBA was added to the solution, RDMB degradation proceeded noticeably slower (Figure S6a), and the *k* decreased from 0.219 to 0.050 min^−1^ (Figure S6b), signifying that ^•^OH shall occur during RDMB elimination. Next, as MeOH was used, the RDMB elimination was substantially inhibited with *k* = 0.006 min^−1^, signifying that SO_4_^•−^ and ^•^OH co-exist in RDMB elimination. Moreover, benzoquinone (BQ) was adopted to examine superoxide (O_2_^•−^). Figure S6a reveals that BQ inhibited RDMB elimination with *k* = 0.034 min^−1^, demonstrating that O_2_^•−^ might be present in RDMB elimination. Moreover, NaN_3_ was used to study singlet oxygen (^1^O_2_), and NaN_3_ also inhibited RDMB elimination with *k* = 0.024 min^−1^, revealing that ^1^O_2_ is also produced from CuCoNC+Oxone.

The occurrence of these radicals at different reaction times were then determined [51,52]. In particular, Figure S6c shows the variations of SO_4_^•−^ and ^•^OH as a function of the reaction time. Nevertheless, the concentration of SO_4_^•−^ seemed much higher than that of ^•^OH, which agrees with the above-mentioned discussions.

Further, the occurrence of ^1^O_2_ was associated with furfuryl alcohol (FFA) consumption [53]. Figure S6c reveals the FFA oxidation by using Oxone alone and CuCoNC+Oxone. Oxone alone seemed to barely lead to FFA consumption, whereas CuCoNC+Oxone led to significant oxidation of FFA, suggesting that CuCoNC would cause the consumption of Oxone and promote production of ^1^O_2_.

Additionally, electron spin resonance (ESR) was employed here to measure radicals species in RDMB degradation. Firstly, when 5,5-Dimethyl-1-pyrroline N-oxide (DMPO) was adopted, no distinct feature was observed in the case of Oxone alone (Figure S6d). When DMPO, Oxone, and CuCoNC were added simultaneously, a noticeable result was produced and attributed to DMPO-SO_4_ and DMPO-OH, as displayed in Figure S6e, validating the existence of SO_4_^•−^ and ^•^OH. Moreover, the same test was then implemented in MeOH to examine whether O_2_^•−^ was present (Figure S6e), and a notable signal was observed and ascribed to DMPO-O_2_.

On the other hand, as another trapping agent 2,2,6,6-tetramethylpiperidine (TEMP) was used, no notable signal was seen for Oxone alone. Once TEMP, Oxone, and CuCoNC were all present, a strong signal was observed and ascribed to TEMP-^1^O_2_ (Figure S6e), confirming that ^1^O_2_ existed.

### 3.6. Decomposition Process of RDMB

**Figure 10:**
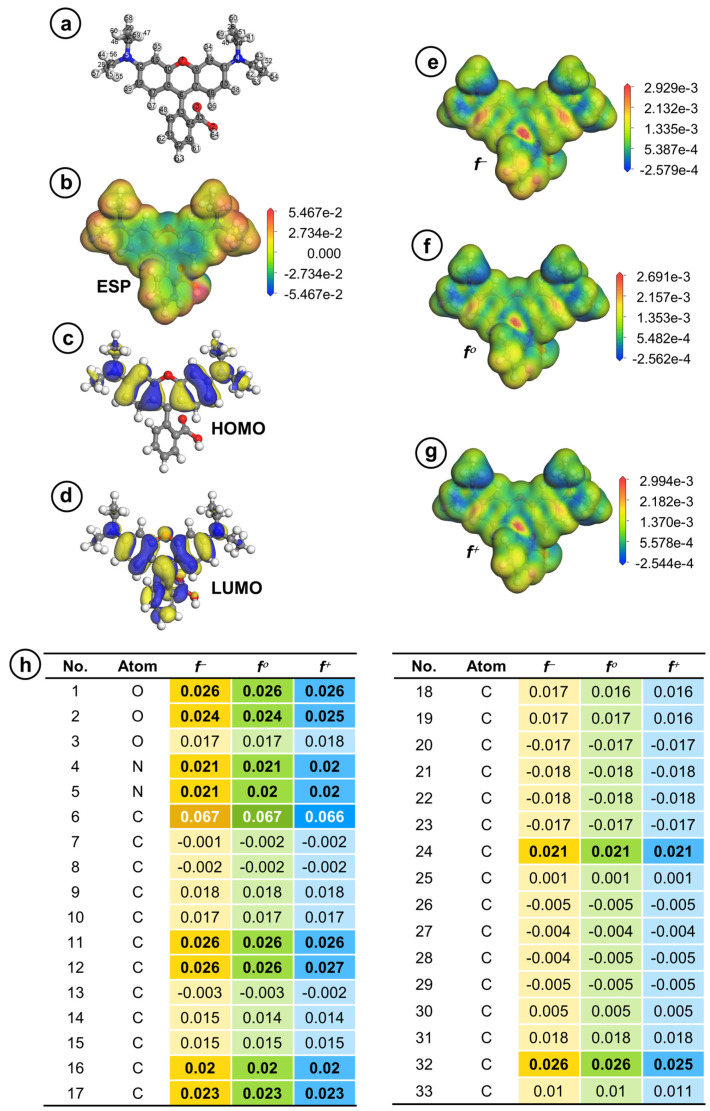
(**a**) Optimized structure; (**b**) ESP; (**c**) HOMO; (**d**) LUMO; (**e**–**g**) electron density mapped by (**e**) *f^−^*, (**f**) *f^ο^*, and (**g**) *f^+^*; and (**h**) Fukui indexes of RDMB molecule (isosurface value = 0.02 electrons/Å^3^).

As CuCoNC can promote Oxone activation to eliminate RDMB, it was essential to study the RDMB elimination process. To this end, the First-Principle-based calculation was employed here [54,55]. In Figure 10a, the optimal configuration of RDMB is calculated and afforded. Specifically, Figure 10b,c reveals the HOMO and LUMO of RDMB. Generally, HOMO regions are relatively susceptible to electrophilic degradation. As SO_4_^•−^, ^•^OH, and ^1^O_2_ would be electrophilic species, the HOMO at the xanthene group of RDMB would be the most susceptible to electrophilic degradation by these species. Figure 10d depicts the ESP distribution of RDMB, indicating that the electron-rich area of RDMB is also relatively accessible to electrophilic degradation.

The Fukui values of RDMB are also generated and summarized in Figure 10e–h for determining potential sites of RDMB for receiving attacks. In general, *f* ^–^, *f* ^0^, and *f* ^+^ indicate the electrophilic, radical, and nucleophilic attacks, separately. As ^1^O_2_ is an electrophilic molecule, fractions of RDMB with larger *f*-values would be more susceptible to electrophilic degradation. Thus, 6C exhibits the highest *f*-values, indicating that 6C might be the initial site for receiving electrophilic degradation (as seen in Figure 10e). In addition, fractions of RDMB with high *f* ^0^ values are also more susceptible to radical degradation; similarly, 6C might be more susceptible to radical degradation during RDMB elimination (as seen in Figure 10f).

**Figure 11:**
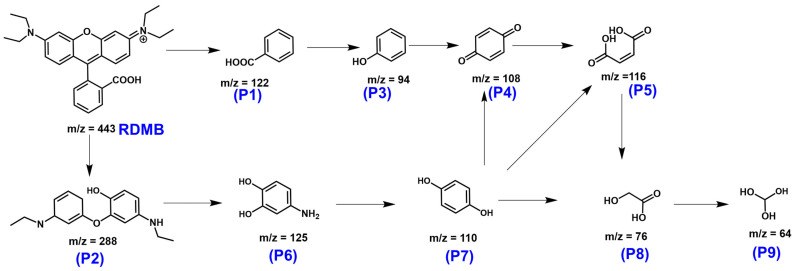
A potential degradation pathway of RDMB degradation by CuCoNC-activated Oxone.

To further examine the degradation process of RDMB using CuCoNC+Oxone, the mass spectrometry of RDMB elimination (Figure S7 and Table S5) was then analyzed and studied with the computational insights to deduce the elimination route in Figure 11. Initially, the bonding between the xanthene ring and benzoic acid group of RDMB received initial attacks that caused the breakage of RDMB, forming P1 and P2 intermediates. P2 would then undergo further oxidation to produce a series of intermediates, such as P3, P4, and P5. Next, P2 was degraded to afford P6, which would then evolve upon oxidation to become P7. P5 and P7, subsequently, would be further broken down to afford a few intermediates, including P8 and P9, finally to CO_2_ and H_2_O.

## 4. Conclusions

In this study, CuCoNC was fabricated to reveal a special hollow-configured morphology and Cu/Co using the template of Co-ZIF followed by the straightforward etching and Cu dopant to afford a practical activator for Oxone. This Cu doping enabled the encapsulation of CuCo into NC, enabling CuCoNC to possess enhanced porous structures, more active sites, and strengthened electrochemistry. Thus, CuCoNC showed much higher catalytic activities than CoNC for Oxone activation to eliminate RDMB. In addition, CuCoNC+Oxone exhibited a lower activation energy for RDMB elimination and retained its effectiveness in eliminating RDMB. These results render CuCoNC a promising activator for Oxone to degrade RDMB.

## Data Availability

Data sharing not applicable.

## Supplementary Materials

The supplementary material can be downloaded at: https://www.mdpi.com/article/10.3390/nano13182565/s1. Table S1. Fractions of N species in catalysts. Table S2. Ratios of *I_D_*/*I_G_* in catalysts. Table S3. A comparison of activation energy (*E_a_*) between CuCoNC+Oxone and other processes for RDMB degradation. Table S4. A comparison between CuCoNC+Oxone and other processes for RDMB degradation. Table S5. Detected by-products of RDMB degradation by CuCoNC+Oxone. Figure S1. (a) SEM, (b) TEM, and (c) EDS of CoNC. Figure S2. (a) SEM image and (b) textural properties of commercial Co_3_O_4_ NP. Figure S3. XPS spectroscopy of CoNC: (a) C1s (b) Co2p, and (c) N1s. Figure S4. zeta potential of CuCoNC. Figure S5. The XRD pattern of used CuCoNC. Figure S6. ESI mass spectra of (a) RDMB and (b) intermediates of RDMB degradation. Figure S7. (a) Effects of various scavengers on RDMB degradation (initial concentration of RDMB = 10 mg/L) and (b) corresponding rate constants; EPR analyses: (c) DMPO, (d) DMPO in methanol, (e) TEMP, (f) Concentrations of ROS; and FFA consumption by CuCoNC+Oxone. (References [56,57,58,59,60,61,62,63] are cited in the supplementary materials).
