# Supplementary material for: Hollow-Architected Heteroatom-Doped Carbon-Supported Nanoscale Cu/Co as an Enhanced Magnetic Activator for Oxone to Degrade Toxicants in Water
Source: Nanomaterials (Basel). 2023 Sep 15;13(18):2565. doi: 10.3390/nano13182565 (PMC10537558; doi:10.3390/nano13182565)
Supplement: Supplementary file 1 [file nanomaterials-13-02565-s001.zip › nanomaterials-2530243-supplementary.pdf]

Supplementary Materials

for

Hollow-Architected Heteroatom-Doped Carbon-Supported Nanoscale Cu/Co as an Enhanced Magnetic Activator for Oxone to Degrade Toxicants in Water

Text S1: Experimental Supplementary Materials

Chemicals and Reagents

Chemical reagents involved in this study were purchased and used as received without additional purifications. Cobalt nitrate hexahydrate ($Co(NO_3)_2.6H_2O$) and $Cu(NO_3)_2$ were obtained from TCI Chemicals (Japan). *h*-methylimidazole (HMIM) (99%) was purchased from Acros Organics (USA). Rhodamine B (RDMB), Oxone ($KHSO_5$), cetyltrimethylammonium bromide (CTAB), tannic acid, methanol, ethanol, *tert*-butanol, sodium azide (NaN_3) were received from Sigma-Aldrich (USA). Deionized (D.I.) water was prepared up to less than 18 MOhm-cm.

Preparation and characterization of CuCoNC

The preparation process of CuCoNC is illustrated as displayed in Fig. 1. Typically, cobalt nitrate hexahydrate (1 mmol), HMIM (0.05 mol) and CTAB (0.01 mmol) were added into a glassware containing 80 mL of D.I. water [1]. The resulting mixture was vigorously stirred for 1 hour at ambient temperature. Then, the purple precipitate was collected by centrifugation (8000 rpm), washed thoroughly by D.I. water/ethanol and dried at 65 oC for 24 hours to obtain cube-like Co-ZIF. Next, a certain amount of the as-obtained Co-ZIF (i.e., 200 mg) was added into a mixture of D.I water and ethanol (ratio = 1/1 in volume) with tannic acid (i.e., 0.018 mmol) and kept stirring for 10 min to complete etching process.

The resultant material was then collected by centrifugation, washed with ethanol several times, followed by mixing with Cu(NO3)2 in N-hexane under sonication for 2 hours. The Cu-doped Co-ZIF was then recovered and dried in an electric oven at 80 oC overnight, and finally carbonized in a continuous N2 flow at 600 oC for 1 hour to afford nanoscale Cu/Co-bearing N-doped carbon (CuCoNC). For comparison, a nanoscale Co-bearing N-doped carbon (CoNC)

without the Cu dopant was also prepared by following the above-mentioned procedure without the mixing with Cu(NO3)2 in n-hexane.

For characterizations, the appearance of the as-prepared materials was firstly visualized by SEM and TEM (JEOL JSM-7800 F and JEM-1400, Japan). The crystalline structure of CuCoNC was also verified by an X-ray diffractometer (Bruker, USA). Moreover, Raman spectroscopy of CuCoNC was analyzed by a Raman spectrometer (Nanofinder 30, Tokyo Instruments, Japan). Besides, the chemical states of elements in CuCoNC was also characterized by X-ray photoelectron spectroscopy (XPS) (PHI 5000, ULVAC-PHI, Japan). Additionally, the physical properties of CuCoNC was analyzed by a volumetric analyzer (Anton Paar Auto IQ, Austria).

Catalytic activation of Oxone for RDMB degradation

The catalytic degradation of RDMB by CuCoNC activated Oxone was conducted using batch experiments. Typically, a small amount of CuCoNC (i.e., 50 mg/L) was firstly added into a RDMB solution with the initial RDMB concentration (C0) of 5 mg/L for 15 min to verify whether RDMB might be adsorbed on CuCoNC surface. Next, 100 mg/L of Oxone was quickly introduced into RDMB solution to initiate the degradation experiment. At a fixed reaction time (t), sample aliquots were withdrawn from the RDMB solution, which were then filtrated by filters to separate CuCoNC from the solution. The remaining RDMB concentration of the filtrate at t (min) (Ct) was subsequently measured using high-performance liquid chromatography (HPLC) with a UV-vis detector at 554 nm. For experiments, the effects of different CuCoNC dosages, Oxone dosages, RDMB initial concentrations, temperatures, initial pH values, co-existing substances and inhibitors were investigated. To explore the reactive radical species contributed to the degradation of RDMB by CuCoNC+Oxone, electron paramagnetic resonance (EPR) was particularly employed using 5,5-Dimethyl-1-pyrroline N-oxide

(DMPO) as a radical spin-trapping agent. The reusability of CuCoNC for continuous Oxone activation to degrade RDMB was further performed by re-using CuCoNC to multiple RDMB degradation cycles. For the recycle experiments, the used CuCoNC was collected via centrifugation, washed thoroughly by D.I. water several times before drying in an electric oven at 65 oC for 24 h, which is then employed for the subsequent degradation experiments. The intermediate products generated from RDMB degradation were also determined by a mass spectrometer (Thermo Finnigan Corporation, LCQ ion-trap mass spectrometer, USA). Moreover, as reactive oxygen species (ROS) might be generated during the activation and involved in RDMB degradation, these species were necessarily determined by electron paramagnetic resonance (EPR) using 5,5-Dimethyl-1-pyrroline N-oxide (DMPO) and 2,2,6,6-tetramethylpiperidine (TEMP) as radical spin-trapping agents to reveal the mechanism of RDMB degradation. Besides, the intermediates derived from RDMB degradation were also determined using a mass spectrometer (Thermo Finnigan Corporation, mass spectrometer, USA).

DFT calculation

The active sites on the RDMB molecule were unraveled using electrostatic Potential (ESP) and Fukui indexes (f^-, f^0, f^+) performed with DFT calculations in the DMol^3 module. The exchange correlation functional of generalized gradient approximation (GGA) and the correction method of Perdew Burke Ernzerhof (PBE) were applied with spin unrestricted and formal spin as initial [16,17]. The double numerical atomic orbital augmented by a polarization function (DNP) was employed as the basis set (basis file was 3.5). For an accurate result, the orbital occupancy was set as using smearing (0.005 Ha) to speed up electronic convergence. The convergence tolerance of energy, threshold of force, max displacement, and orbital cutoff were set to 2.0×10^{-5} Ha, 0.004 Ha/Å, 0.005 Å, and 4.6 Å, respectively.

Table S1. Fractions of N species in catalysts

Species	*CuCoNC*	*CoNC*
Pyridinic N	*37.0%*	*48.1%*
Pyrrolic N	*45.3%*	*19.9%*
Quaternary N	*17.7%*	*22.1%*
N-O	-	*9.9%*

Table S2. Ratios of I_D/I_G in catalysts

Ratio	*CuCoNC*	*CoNC*
I_D/I_G	*1.26*	*0.82*

Table S3. A comparison of activation energy (E_a) between CuCoNC+Oxone and other processes for RDMB degradation

Catalyst	E_a (kJ /mol)	Ref.
**CuCoNC**	***27.7***	This study
**α-MnO_2**	*38.2*	[1]
**$Cu/Al_2O_3/g\text{-}C_3N_4$**	71.0	[2]
**Carrot-derived carbon**	35.9	[3]
**Cu complex on silica**	38.6	[4]
**Silica supported-iron**	82.35	[5]
**$CoFe_2O_4$/TNT**	*70.6*	[6]

Table S4. A comparison between CuCoNC+Oxone and other processes for RDMB degradation

Catalyst	Pollutants	$C_{pollutant}$	Optimum Conditions				Max Removal (%)	Ref.
			pH	Oxone dose	Catalyst dose (mg/L)	Reaction time (min)		
CuCoNC	Rhodamine B	5 mg/L	-	100 mg/L	50 mg/L	30 min	93	This work
Co_3O_4	Rhodamine B	5 mg/L	-	100 mg/L	50 mg/L	30 min	68	This work
CoNC	Rhodamine B	5 mg/L	-	100 mg/L	50 mg/L	30 min	89	This work
Co/SBA-15	Rhodamine B	5 mg/L	-	Oxone:Rhodamine B 10:1	0.1 g/L	120 min	99	[7]
10Co10Mg/SBA-15	Rhodamine B	5 mg/L	-	Oxone:Rhodamine B 10:1	0.1 g/L	7 min	96	[7]
$Co_{0.75}Fe_{2.25}O_4$ NPs	Rhodamine B	0.014 mmol/L	6.0	1.0 mmol/L	50 mg/L	40 min	99	[8]
ZIF67	Rhodamine B	50 mg/L	-	150 mg/L	10 mg/L	60 min	80	[9]
$LaCoO_3/ZrO_2$	Rhodamine B	10 mg/L		100 mg/L	100 mg/L	60 min	99	[10]
CoMgFe-LDH	Rhodamine B	90 µM	5.0	1.2 mM	0.8 g/L	10 min	94.3	[11]
JXUST-23	Rhodamine B	100 mg/L	9.0	100 mg/L	100 mg/L	60 min	91.5	[12]

Table S5. Detected by-products of RDMB degradation by CuCoNC+Oxone

	Structure		Structure
RDMB	Chemical Formula: $C_{28}H_{31}N_2O_3^+$<br>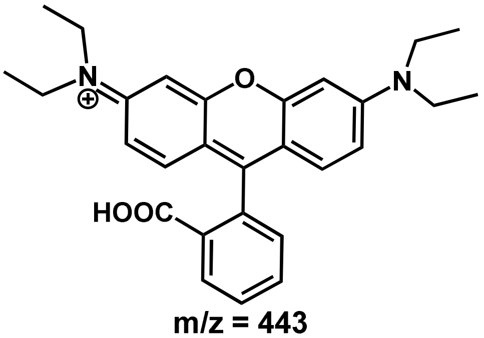<br>**m/z = 443**	P5	Chemical Formula: $C_4H_4O_4$<br>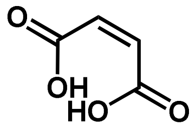<br>**m/z =116**
P1	Chemical Formula: $C_7H_6O_2$<br>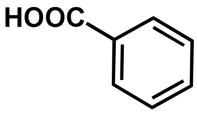<br>**m/z = 122**	P6	Chemical Formula: $C_6H_7NO_2$<br>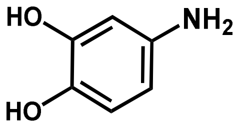<br>**m/z = 125**
P2	Chemical Formula: $C_{17}H_{24}N_2O_2$<br>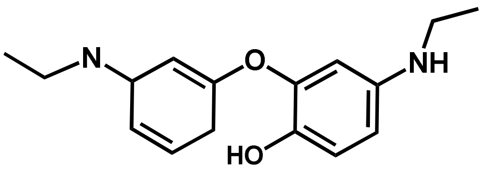<br>**m/z = 288**	P7	Chemical Formula: $C_6H_6O_2$<br>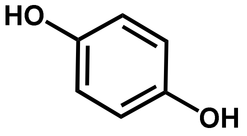<br>**m/z = 110**
P3	Chemical Formula: C_6H_6O<br>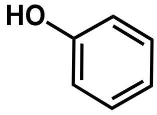<br>**m/z = 94**	P8	Chemical Formula: $C_2H_4O_3$<br>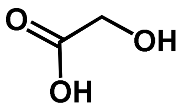<br>**m/z = 76**
P4	Chemical Formula: $C_6H_4O_2$<br>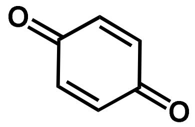<br>**m/z = 108**	P9	Chemical Formula: CH_4O_3<br>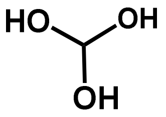<br>**m/z = 64**

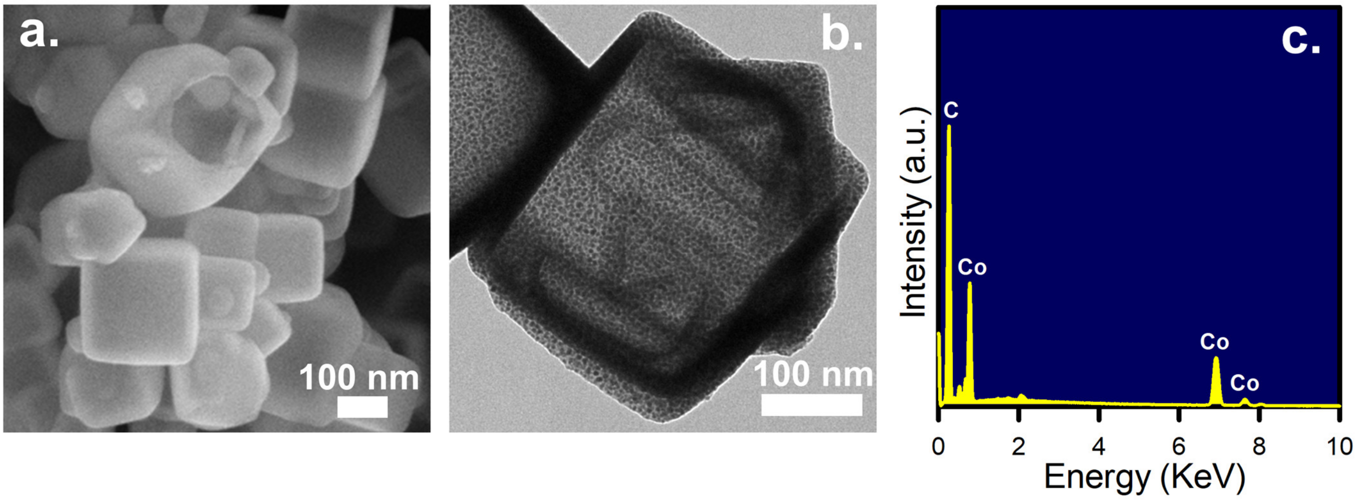

Figure S1. (a) SEM, (b) TEM, and (c) EDS of CoNC

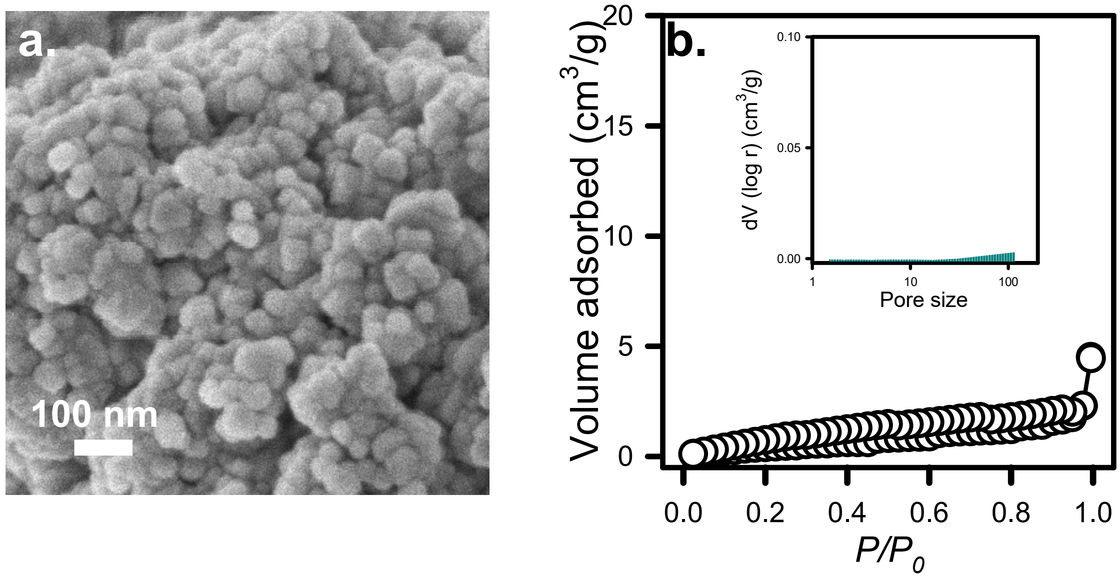

Figure S2. (a) SEM image and (b) textural properties of commercial Co_3O_4 NP.

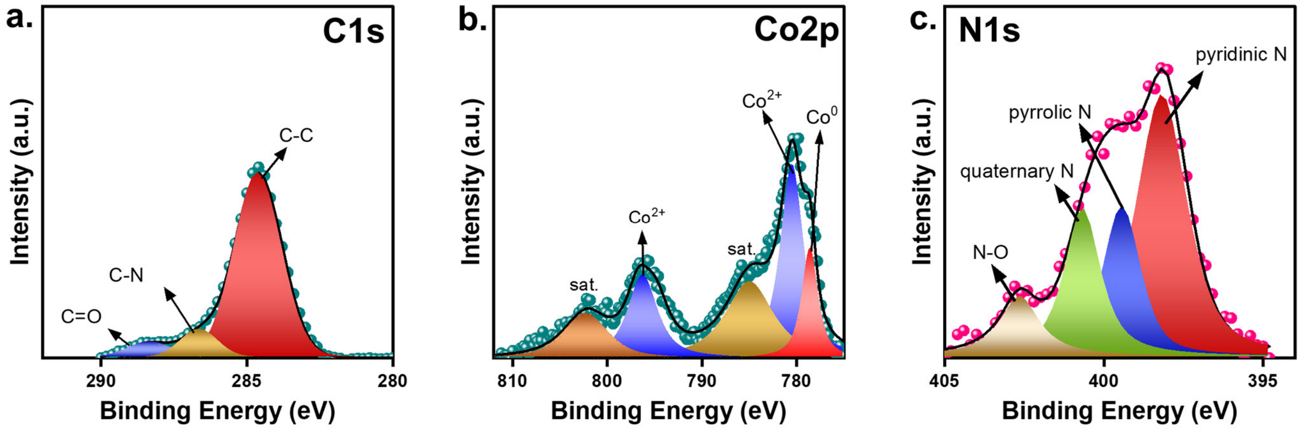

Figure S3. XPS spectroscopy of CoNC: (a) C1s (b) Co2p, and (c) N1s.

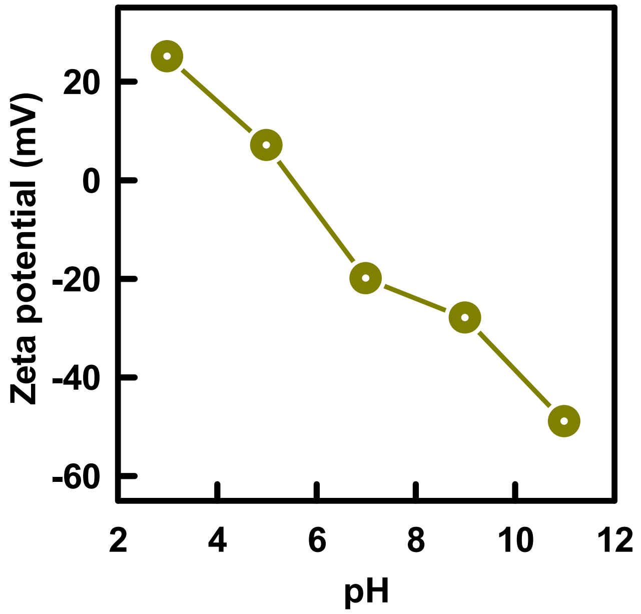

Figure S4. zeta potential of CuCoNC

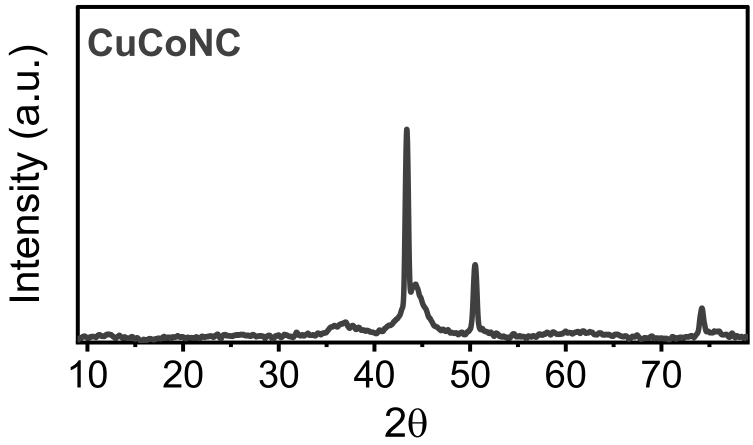

Figure S5. The XRD pattern of used CuCoNC.

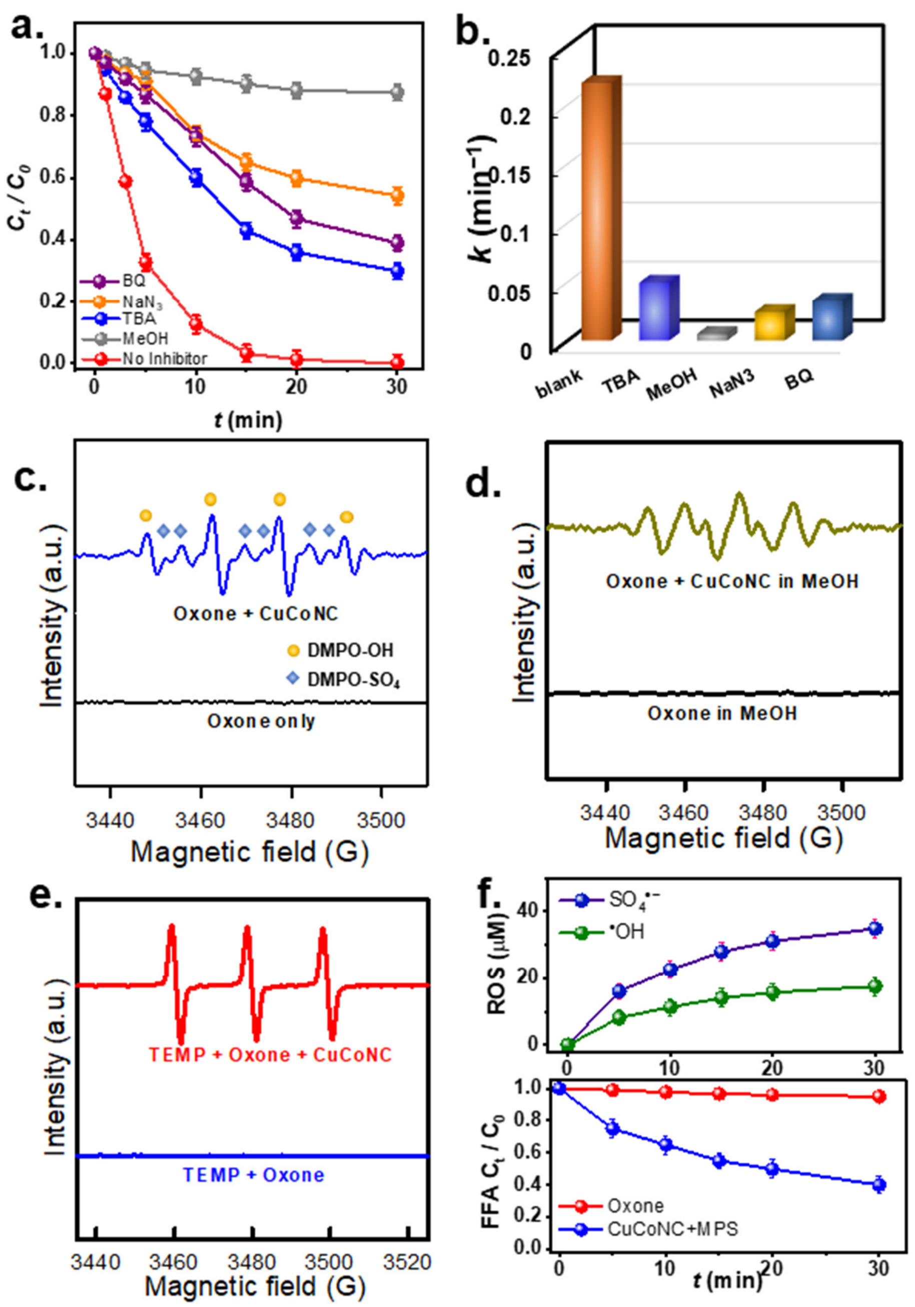

Figure S7. (a) Effects of various scavengers on RDMB degradation (initial concentration of RDMB = 10 mg/L) and (b) corresponding rate constants; EPR analyses: (c) DMPO, (d) DMPO in methanol, (e) TEMP, (f) Concentrations of ROS; and FFA consumption by CuCoNC+Oxone.

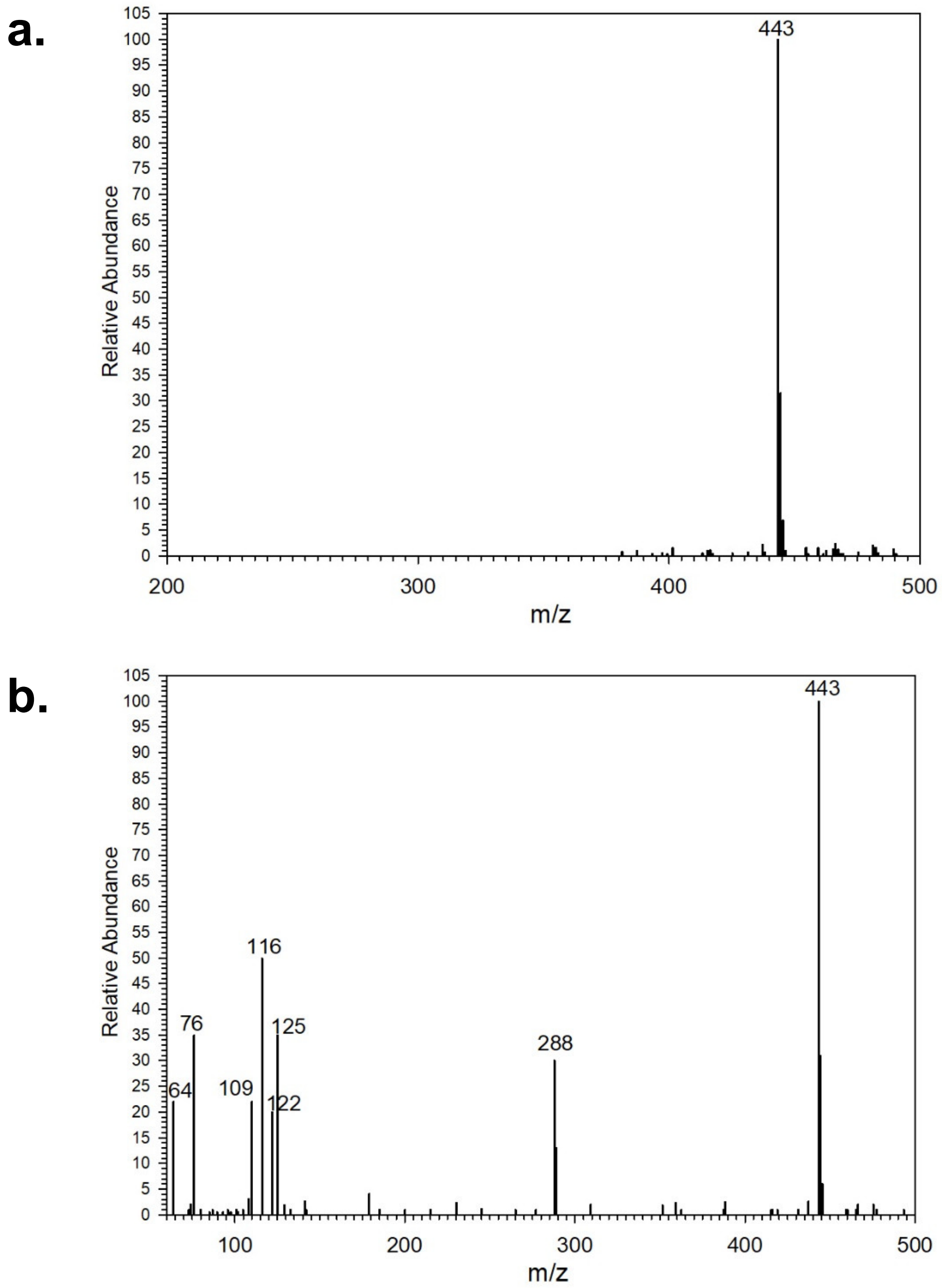

Figure S6. ESI mass spectra of (a) RDMB and (b) intermediates of RDMB degradation.

References:

[1] C. Huang, Y. Wang, M. Gong, W. Wang, Y. Mu, Z.-H. Hu, α-MnO2/Palygorskite composite as an effective catalyst for heterogeneous activation of peroxymonosulfate (PMS) for the degradation of Rhodamine B, Separation and Purification Technology, 230 (2020).

[2] C. Zhou, Z. Liu, L. Fang, Y. Guo, Y. Feng, M. Yang, Kinetic and Mechanistic Study of Rhodamine B Degradation by H2O2 and Cu/Al2O3/g-C3N4 Composite, Catalysts, 10 (2020) 317.

[3] S.A. Hira, M. Yusuf, D. Annas, H.S. Hui, K.H. Park, Biomass-Derived Activated Carbon as a Catalyst for the Effective Degradation of Rhodamine B dye, Processes, 8 (2020) 926.

[4] W. Yuan, C. Zhang, H. Wei, Q. Wang, K. Li, In situ synthesis and immobilization of a Cu(ii)–pyridyl complex on silica microspheres as a novel Fenton-like catalyst for RhB degradation at near-neutral pH, RSC Adv, 7 (2017) 22825-22835.

[5] P.P. Gan, S.F.Y. Li, Efficient removal of Rhodamine B using a rice hull-based silica supported iron catalyst by Fenton-like process, Chemical Engineering Journal, 229 (2013) 351-363.

[6] Y. Du, W. Ma, P. Liu, B. Zou, J. Ma, Magnetic CoFe2O4 nanoparticles supported on titanate nanotubes (CoFe2O4/TNTs) as a novel heterogeneous catalyst for peroxymonosulfate activation and degradation of organic pollutants, J Hazard Mater, 308 (2016) 58-66.

[7] L. Hu, F. Yang, W. Lu, Y. Hao, H. Yuan, Heterogeneous activation of oxone with CoMg/SBA-15 for the degradation of dye Rhodamine B in aqueous solution, Applied Catalysis B: Environmental, 134 (2013) 7-18.

[8] S. Su, W. Guo, Y. Leng, C. Yi, Z. Ma, Heterogeneous activation of Oxone by CoxFe3− xO4 nanocatalysts for degradation of rhodamine B, Journal of Hazardous Materials, 244 (2013) 736-742.

[9] K.-Y.A. Lin, H.-A. Chang, Zeolitic Imidazole Framework-67 (ZIF-67) as a heterogeneous catalyst to activate peroxymonosulfate for degradation of Rhodamine B in water, Journal of the Taiwan Institute of Chemical Engineers, 53 (2015) 40-45.

[10] K.-Y.A. Lin, Y.-C. Chen, T.-Y. Lin, H. Yang, Lanthanum cobaltite perovskite supported on zirconia as an efficient heterogeneous catalyst for activating Oxone in water, Journal of colloid and interface science, 497 (2017) 325-332.

[11] G. Liu, Y. Liu, D. Chen, C. Wang, W. Guan, Activation of peroxymonosulfate by Co-Mg-Fe layered doubled hydroxide for efficient degradation of Rhodamine B, Environmental Science and Pollution Research, 30 (2023) 37634-37645.

[12] R.-J. Zhang, B.-L. Chai, H. Xu, T.-F. Zheng, Z.-H. Zhu, Y. Peng, J.-L. Chen, S.-J.

Liu, H.-R. Wen, Enhanced Heterogeneous Catalytic Activity of Peroxymonosulfate for Rhodamine B Degradation via a CoII-Based Metal–Organic Framework, Inorganic Chemistry, 62 (2023) 2760-2768.
